# Automated Bayesian model development for frequency detection in biological time series

**DOI:** 10.1186/1752-0509-5-97

**Published:** 2011-06-24

**Authors:** Emma Granqvist, Giles ED Oldroyd, Richard J Morris

**Affiliations:** 1 Department of Computational & Systems Biology, John Innes Centre, Norwich Research Park, Norwich NR4 7UH, UK; 2 Department of Disease & Stress Biology, John Innes Centre, Norwich Research Park, Norwich NR4 7UH, UK

## Abstract

**Background:**

A first step in building a mathematical model of a biological system is often the analysis of the temporal behaviour of key quantities. Mathematical relationships between the time and frequency domain, such as Fourier Transforms and wavelets, are commonly used to extract information about the underlying signal from a given time series. This one-to-one mapping from time points to frequencies inherently assumes that both domains contain the complete knowledge of the system. However, for truncated, noisy time series with background trends this unique mapping breaks down and the question reduces to an inference problem of identifying the most probable frequencies.

**Results:**

In this paper we build on the method of Bayesian Spectrum Analysis and demonstrate its advantages over conventional methods by applying it to a number of test cases, including two types of biological time series. Firstly, oscillations of calcium in plant root cells in response to microbial symbionts are non-stationary and noisy, posing challenges to data analysis. Secondly, circadian rhythms in gene expression measured over only two cycles highlights the problem of time series with limited length. The results show that the Bayesian frequency detection approach can provide useful results in specific areas where Fourier analysis can be uninformative or misleading. We demonstrate further benefits of the Bayesian approach for time series analysis, such as direct comparison of different hypotheses, inherent estimation of noise levels and parameter precision, and a flexible framework for modelling the data without pre-processing.

**Conclusions:**

Modelling in systems biology often builds on the study of time-dependent phenomena. Fourier Transforms are a convenient tool for analysing the frequency domain of time series. However, there are well-known limitations of this method, such as the introduction of spurious frequencies when handling short and noisy time series, and the requirement for uniformly sampled data. Biological time series often deviate significantly from the requirements of optimality for Fourier transformation. In this paper we present an alternative approach based on Bayesian inference. We show the value of placing spectral analysis in the framework of Bayesian inference and demonstrate how model comparison can automate this procedure.

## Background

Pattern recognition is central to many scientific disciplines and is often a first step in building a model that explains the data. In particular, the study of periodic phenomena and frequency detection has received much attention, leading to the well-established field of spectral analysis.

Biology is rich with (near) periodic behaviour, with sustained oscillations in the form of limit cycles playing important roles in many diverse phenomena such as glycolytic metabolism, circadian rhythms, mitotic cycles, cardiac rhythms, hormonal cycles, population dynamics, epidemiological cycles, etc. [1]. A conventional method for frequency detection is Fourier analysis. It is based on the fact that it is possible to represent any integrable function as an infinite sum of sines and cosines. The Fourier Transform (FT) uses this property to reveal the underlying components that are present in a signal [2]. Fourier theory has given rise to a wide range of diverse developments and far-reaching applications, demonstrating the theory's undisputed importance and impact. For frequency detection, however, it is known that the FT works optimally only for uniformly sampled, long, stationary time series. Furthermore, common procedures of pre-processing the data can cause problems. Time series can contain low frequency background fluctuations or drift that are unrelated to the signal of interest. For the FT, it is then necessary to remove the trends using detrending techniques. As has been shown previously, this detrending leads to convolution of the signal that can both remove evidence for periodicity and add false patterns [3]. Another known problem is aliasing. If a signal containing high frequencies is recorded with a low sampling rate, peaks of high frequencies can fold back into the frequency spectrum, appearing as low frequencies [2]. The Gibbs phenomenon provides another example where spurious peaks appear in a FT. It occurs at points of discontinuity in a periodic function, and results in so-called ringing artefacts around the "true" frequency peak [4]. As for the accuracy of the frequency estimate, no direct information of this is given by the output from a FT, since the sharpness of the peaks depends on a combination of factors such as noise levels and the length of the time series. For further details, see the extensive FT literature (e.g. [2,5]).

Wavelet Transforms [6-10] offer an attractive alternative to Fourier Transforms. The main difference is that they are localised in both the time and frequency domain. This property makes wavelets better adapted to problems with truncated data. Wavelets have found wide-ranging applications and have proven to be particularly powerful for image processing and data compression [11-13].

Bayesian inference provides another approach for analysing data (for an introduction to Bayesian analysis, see [14]). It addresses additional aspects of the problem, such as the inherent uncertainty of the analysis and the effects of external noise. Using this framework [3], the method of Bayesian Spectrum Analysis (BSA) was developed by Bretthorst [15] and applied to Nuclear Magnetic Resonance (NMR) signals and parameter estimation with great success [16,17].

There are several advantages of the Bayesian approach, including an inherent mechanism for estimating the accuracy of the result and all parameters, as well as the ability to compare different hypotheses directly. Focus is shifted to the question of interest by integrating out other parameters and noise levels. Initial knowledge of the system can be incorporated in the analysis and expressed in the prior probability distributions. There has been a recent flood of Bayesian papers with some convincing applications and promising developments in systems biology (see [18-30], and many others). The Bayesian approach to time series analysis has proven its value in fields such as NMR and ion cyclotron resonance analysis (e.g. [31] and [32]).

In this paper, we describe the development, implementation and testing of Bayesian model development coupled with BSA and Nested Sampling, in a biological context. We present a comparison of this approach with the FT, applied to a number of simulated test cases and two types of biological time series that present challenges to accurate frequency detection. We first present some necessary background, upon which we build to develop to our approach.

### Bayesian inference

Data is rarely available in sufficient quantity and quality to allow for exact scientific deduction. Instead we are forced to infer models from incomplete knowledge. Bayesian inference is based on Bayes' Rule, which evaluates a hypothesis, *H*, in light of some data, *D*, and some prior information, *I*. It is a method of assigning probabilities based on the current state of knowledge, allowing for subsequent re-evaluation as new data becomes available. The goal is to determine *P *(*H *|*D*, *I*), the posterior probability distribution of the hypothesis, given the data and the prior information. With Bayes' rule, the posterior can be expressed in terms of other probabilities as(1)

where *P*(*D*|*H*, *I*) is the probability of observing the data given the hypothesis and the prior, *P*(*H*|*I*) is the prior probability of the hypothesis, and *P*(*D*|*I*) is the probability of the data given the prior. When the hypothesis is the variable and the data is held constant, *P*(*D*|*H*, *I*) is called the likelihood function, and when the hypothesis is constant it is called the probability of obtaining a specified outcome (data). When evaluating only one hypothesis, *P*(*D*|*I*) is a normalising constant, but when investigating more than one hypothesis this term plays a key role and is called the evidence [33].

### Bayesian Spectrum Analysis

Our presentation in this section follows closely that of Bretthorst [15]. The aim is to infer the most probable frequency (or frequencies), ***ω***, from the given data. We start by building a model (the hypothesis *H*) for the observed data, parameterised by angular frequency, ***ω***, and amplitudes, **c**, and then use Bayes' rule to compute the posterior probability of the parameters, *P*(***ω***, **c**|*D*, *H*, *I*). By assigning priors to the model parameters **c **and integrating over these, we arrive at the posterior probability for the parameter of interest, ***ω***, . This is referred to as the marginal posterior probability of ***ω***. We note that ***ω ***is an r-tuple, {*ω*_1_, *ω*_2_, ..., *ω_r_*}, with as many elements as there are distinct frequencies in the data.

A general model for observed data sampled at *N *discrete time points, *D *= {*d*(*t*_1_), ..., *d*(*t_N_*)}, includes the signal of interest, *s*(*t_i_*), a possible background function, *g*(*t_i_*), and the noise present in the system, *e*(*t_i_*),(2)

The signal function will usually be unknown and may be complicated, but can be approximated by a linear combination of *m*_s_ model functions, *ψ_i_*, that we parameterise by the quantity of interest, ***ω***:(3)

in which  are the expansion coefficients.

Similarly, the background function, *g*(*t_i_*), can be approximated by a set of *m_g _*functions, *ζ_i_*, that are independent of ***ω***,(4)

where  are the background model function expansion coefficients.

Since **a **and **b **are not the main focus of the analysis, we will aim to integrate them out of the equations by marginalisation. Parameters that are treated in this manner as often referred to as nuisance parameters, which we denote here by . Although the signal function depends on ***ω***, whereas the background function does not, for notational purposes we introduce the set of model functions, *ϕ_i_*, which consists of both *ψ_i _*and *ζ_i_*. This allows us to condense the model equation into(5)

such that now *d*(*t_i_*) = *f*(*t_i_*) + *e*(*t_i_*) and *m *is now the total number of model functions, *m_s _*+ *m_g_*. The model functions will typically not be orthogonal functions over the time series domain. This, however, can be achieved by Cholesky decomposition. In all subsequent calculations an orthogonal basis is used. From Bayes' rule, the joint probability distribution of the model for the parameters ***ω ***and **c **is(6)

The likelihood function, *P*(*D*|***ω***, **c**, *H*, *I*), is calculated by comparing data produced by the model signal, equation (5), to real experimental data. If the model perfectly captures the signal, the difference between the model data and the real data is simply the noise in the system. The model of the data in equation (2) includes noise, *e*(*t_i_*), which we assume to be time independent in the further developments. The true noise level is unknown, but for a given noise power, *σ*^2^, the principle of maximum entropy leads to the use of a normal distribution,(7)

A noise model of this form ensures that the accuracy of the results is maximally conservative for a given noise power. We will later integrate over all possible noise levels to remove the dependence on *σ*. With the described signal and this noise model, the likelihood was calculated by Bretthorst [15] to be(8)

where *N *is the number of data points, and(9)

where  is the mean-square of the data, , and Φ*_jk _*is the matrix of the model functions, .

The goal of the analysis is to compute the posterior probability for frequencies in the data, i.e. to go from the joint probability distribution to a posterior probability of ***ω***, independent of the other parameters. By integrating over all possible values of the parameters *σ *and **c**, the remainder is the marginal posterior of the parameters of interest, ***ω***={*ω*_1_, *ω*_2_, ..., *ω_r_*}. This is an essential advantage of the Bayesian framework, allowing the analysis to focus on estimating the parameters of interest, regardless of the values of the others. If necessary, the other parameters can be estimated at a later point.

To integrate over the σ and **c **values, priors must first be assigned to them. We chose uniform priors for **c **and ***ω***, representing complete lack of knowledge. We know that *σ *is continuous and must be positive, and in such cases a Jeffreys prior is appropriate, *P*(*σ*|*I*) = 1/*σ*. Both the uniform distribution over continuous variables and the Jeffreys prior are known as improper priors if bounds are not specified as they cannot be normalised. For more information on prior assignment see [3,15].

Using the general model, equation (5), assigning the priors, calculating the likelihood function, equation (8), and integrating out the amplitudes and noise parameters, the posterior probability distribution of ***ω ***is proportional to(10)

where *h *is the projection of the data onto the orthonormal model functions, , and  is the mean-square of the *h_j_*, , [15]. This expression of the posterior allows us to identify the strongest frequencies present in the data. For a good model, there will be a high probability peak in the posterior distribution at that **ω **= {*ω*_1_, *ω*_2_, ..., *ω_r_*}.

## Results and Discussion

We employed the framework developed by Jaynes [3] and Bretthorst [15] to investigate the frequency components in a number of biological time series.

### Model comparison

After evaluating the probability of parameters in light of a certain hypothesis, it is important to question the validity of that hypothesis. Thus, the next step in Bayesian inference is to compare the probability of different hypotheses. The hypothesis is now a particular model of the signal, *H_i_*, out of a set of *M *models {*H*_1_, ..., *H_M_*}, and using Bayes' Rule, the posterior probability of this model is(11)

Then two different models, *H_i _*and *H_j_*, can be compared by taking their ratios,(12)

The probability of the data given our prior information, *P*(*D*|*H_i_*, *I*), which was a normalisation constant in equation (6), will now vary between models, and is called the evidence. It evaluates the fit of the data to the model, whilst penalising models that include more parameters. Each additional model parameter should be followed by a significant increase in probability, otherwise the simpler model is preferred. Thus, Bayesian model comparison naturally follows the principle of Occam's razor [33,34].

#### Model development

It will often not be obvious which function to choose to model trends in the data, so an approach using basis functions and expanding these to different orders will be of advantage, as in equation (4). Each expansion represents a different model, *H_i_*, and these can be compared using inference techniques. Likewise, different functions for capturing the signals in the data and modelling a different number of signals correspond to different models for data. Following [3,14,33], we use the posterior ratio to evaluate different models.

This model ratio can be used to determine the number of background model functions for each time series. The posterior probability ratio is calculated between model *H_n _*and *H*_*n*+1_, where *H_n _*is a model including *n *background functions. To obtain the model ratio, priors are assigned to each of the models and their likelihood functions are calculated. Assigning equal prior probability to all models reduces this to the ratio of evidences. To compute the evidences we need to integrate the likelihood, *P*(*D*|***ω***, *σ*, **c**, *H_n_*, *I*) from equation (8), over ***ω***, *σ*, and **c **for each model *H_n_*. By assigning proper normalised priors to all model parameters it is possible to integrate over them around the maximum likelihood estimate. Following Bretthorst's derivation for location parameters [15], we assign Gaussian priors to the amplitudes with hyperparameters for the variances. Since the variances are scale parameters, they are subsequently assigned Jeffreys' priors with an upper and lower bound. This allows us to normalise them and integrate, leaving the defined bounds as parameters in the final equation. For models with the same bounds these terms cancel out in the model ratio. The evidence for a given model, *H_n_*, was calculated by Bretthorst [15] to be(13)

where *δ*, *γ *and *σ *are the prior variances for amplitudes, frequencies and noise, respectively, *R_δ_*, *R_γ _*and *R*_*σ *_are the ratios of the integral bounds for these variances,  is the mean-square projection of the data onto the orthonormal model functions at the maximum likelihood point for model *H_n_*,  is the mean-square of the ***ω ***value that maximises the likelihood, , and *r *is the number of ***ω ***parameters, ***ω ***= {*ω*_1_, ..., *ω_r_*}. The Jacobian  is obtained by orthogonalising the Taylor-expansion of  around the maximum-likelihood point, . See Bretthorst for further details [15]. For cases in which the number of frequencies in the data exceeds the dimension of omega, for instance multiple frequency data with a single frequency model, the above approximation for the evidence is ill-suited as the posterior will cease to be unimodal. For such scenarios, either multiple expansions or MCMC offer attractive solutions to marginalisation. For comparison we have included results from Nested Sampling [14,35] as a means to perform the integration and compute the evidence. Nested Sampling is a variant of MCMC that employs a likelihood based sorting of sample points to efficiently guide the search strategy of the posterior distribution [14,35].

When the model ratio, *H_n_*/*H*_*n*__+__1_, becomes greater than 1, the simpler model, *H_n_*, is favoured over *H*_*n*+1 _[33]. Adding more background functions than are justified by the data (based on the posterior model ratio) may lead to a lower probability for the frequency and in some cases possibly a location shift.

This model development approach used for the background functions above can also be used to decide on the number of underlying frequencies in the data. The model ratios of a time series containing one frequency (case A) and a time series containing two (case B) are presented in Table 1, analysed with both a one- and two-frequency model. The results show, as expected, a preference for the one-frequency model in case A, and for the two-frequency model in case B.

**Table 1:** Model ratios for number of frequencies in the data

Case	*N_ω_*	Models	Model ratio
A	1	*H*_1*ω*_/*H*_2*ω*_	66936
B	2	*H*_1*ω*_/*H*_2*ω*_	0
B	2	*H*_2*ω*_/*H*_3*ω*_	686

Case A has only one frequency, *N_ω _*= 1, and case B has two, *N_ω _*= 2. The model ratio of a model including one frequency, *H*_1*ω*_, and a model with two, *H*_2*ω*_, is well above 1 for case A, showing that the simpler model should be chosen. For case B, the first ratio is close to 0, showing that the more complex model with two frequencies is far more likely. Values below 10^-8 ^are listed as 0. The second ratio is above 1, preferring the model with two frequencies to the one with three.

We point out that the proposed method stops once the current best model has been found but is not guaranteed to find the global maximum from a predefined set of models. The procedure is thus part of model development rather than model selection. If the set of hypotheses are known in advance then the posterior ratios over the full set should be used to find the best model.

### Testing

We first show the power of the BSA approach on test cases using simulated data. In these tests, we sought to recover known input parameters from the simulated data, to validate the BSA approach. We employed sines and cosines as model functions (*ψ_j _*in equation (3)). For comparison, Discrete Fourier Transforms were computed using Fast Fourier Transforms (FFT) [36]. In the test cases, we varied key parameters such as noise levels, trace length, sampling intervals, amplitudes, frequencies, background trends and shape of oscillations.

Representative cases of noise levels and background trends are shown in Table 2, including FFT results on the same data set. A key observation is that the Bayesian approach extracts the correct answer from the data with high precision. BSA also computes the signal-to-noise ratios which is a useful indication of how much of the data cannot be accounted for in the model. Furthermore, the amplitudes do not impact the BSA results since they are integrated out.

**Table 2:** BSA and FFT results from simulated harmonic data with noise and background trends

**No**.	*ω*	*e_a _*(%)	*e_p _*(%)	*b*		*σ_FFT_*		*σ_BSA_*		*σ_BSA-NS_*	s-n
1	0.5	1	-	-	0.49	0.06	0.5	0	0.5	0.0002	70
2	0.5	10	-	-	0.49	0.20	0.5	0.0002	0.5	0.0004	6.5
3	0.5	40	-	-	0.49	0.54	0.5	0.0005	0.5	0.0011	1.9
4	0.5	10	10	-	0.49	0.27	0.5	0	0.5	0.0003	4.2
5	0.5	10	40	-	0.49	0.57	0.5	0.0002	0.5	0.0007	2.2
6	0.5	100	40	-	0.49	0.89	0.5	0.0006	0.5	0.0020	0.7
7	0.3, 0.5	10	10	-	0.29, 0.51	0.14	0.3, 0.5	0.0003	0.34	0.0832	1
8	0.5	10	-	-*t*	0	0.15	0.5	0.0002	0.5	0.0002	110
9	0.5	10	-	-*t*^2^	0	0.19	0.5	0.0002	0.5	0.0002	90
10	0.5	10	-	-*t*^3^	0.02	0.24	0.5	0.0003	0.5	0.0002	35

Each time series was generated with a sine function of angular frequency, *ω*, of 0.5 rad/s with a level of noise in amplitude, *e_a_*, and phase, *e_p_*. In some time series a background trend (*b*) was included, and in case number 7 an additional sine function of 0.3 rad/s is present. The resulting function was sampled 200 times at an interval of 1 s. Results from FFT are presented in the form of the angular frequency with the highest power, , and the estimated standard deviation, *σ_FFT_*. The Bayesian frequency estimate at the maximum posterior point is denoted by  and its standard deviation by *σ_BSA_*. For comparison, the expectation value of *ω *and its standard deviation computed using BSA and Nested Sampling (BSA-NS) are denoted by  and *σ_BSA-NS_*. Values of σ below 10^-8 ^are listed as 0. The estimated signal-to-noise ratio (s-n) from the Bayesian analysis is given in the last column. The BSA and BSA-NS approaches deliver the same results, apart from the case of multiple frequencies in a 1D search of *ω *(case No. 7), which for BSA-NS leads an intermediate estimate between the frequencies with a higher standard deviation.

BSA has a clear advantage over FFT when the data is non-uniformly sampled. FFT requires uniform sampling, whilst BSA is less stringent and delivers the correct result with higher precision. Bretthorst also noted that non-uniformly sampled data removes aliases from the frequency domain, another significant advantage [15]. Five further distinct cases emerged from the tests in which BSA delivers superior results to FFT: time series which have background trends, few data points, high noise levels, multiple frequencies, and non-harmonic oscillations.

#### Background trends

Additional file 1, Figure S1, is an example of a time series with a strong background trend. In Table 3, the model ratios for different background functions are shown. The ratio is initially well below 1, but the ratio of models with expansion orders of two and three Legendre polynomials is above 1. Thus, background functions of Legendre polynomials to expansion order two is more likely, and should be used in the estimation of *ω*.

**Table 3:** Automated model development

Models	Model ratio
*H*_0*ζ *_/*H*_1*ζ*_	1.9459e-06
*H*_1*ζ *_/*H*_2*ζ*_	1.0256e-167
*H*_2*ζ *_/*H*_3*ζ*_	622.5
*H*_3*ζ *_/*H*_4*ζ*_	566.3
*H*_4*ζ *_/*H*_5*ζ*_	501.8
*H*_5*ζ *_/*H*_6*ζ*_	99.2

Posterior probability ratios of models including a different a number of background functions, *ζ*, in the analysis of the time series in Additional file 1, Figure S1. The first two ratios favour the more complex model (ratio below 1). The ratio between models with two and three background functions, *H*_2*ζ *_/*H*_3*ζ*_, is above 1 thus favouring the simpler model. The analysis would then normally automatically stop at two background functions, but for demonstration we include more functions here. The ratio stays above 1 thereby building a chain of decisions that always prefer the simpler model.

Examples 8-10 in Table 2 also include trends, and without pre-processing FFT cannot pick out the correct frequency. In contrast, BSA includes background functions in the model signal and delivers the desired result. Including background functions, however, results in over-estimation of the signal-to-noise ratio.

#### Short time series

Additional file 2, Figure S2, shows the results from analysing a short time series. The FFT power spectrum is very broad (Additional file 2B, Figure S2B), which comes as no surprise given the FFT dependence on the number of data points. BSA estimates the correct frequency sharply, but the maximum probability drops compared to longer time series (Additional file 2C, Figure S2C). This demonstrates the higher uncertainty associated with fewer time points.

#### High noise levels

**Figure 1:**
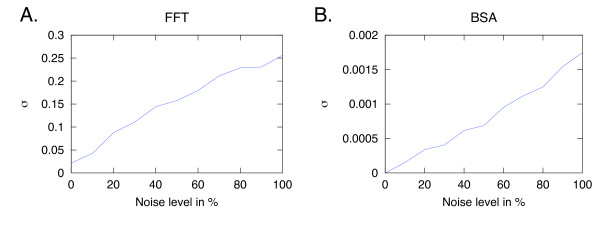
**Effects of noise on precision**. The effect of noise on *σ *for FFT (A) and BSA (B). The time series were simulated from *d*(*t*) = sin(*ωt*) + *e*, with *ω *= 0.5 rad/s and sampled with 3 s intervals to give 100 points. The noise level, *e*, varies between 0 and 100%. Although the qualitative behaviour of noise on the precision is the same for FFT and BSA in this example, BSA produces values of σ that are two orders of magnitude below the FFT values.

BSA is also successful at handling high levels of noise, as highlighted in Examples 1-6 in Table 2. The frequency estimates are correctly reproduced by the FFT. In these simple test scenarios, the BSA posterior probability distribution estimates the frequency with a significantly higher precision than the FFT. Whereas the estimated uncertainty of parameter expectation values is a built-in aspect of any probabilistic treatment such as BSA, FFT has no inherent mechanism for assessing the accuracy of the results. The FFT output is summarised by the average, , and the standard deviation, *σ_FFT_*, over the transformed data set. We show that different noise levels influence the *σ_FFT _*more than the *σ_BSA _*(Figure 1).

#### Multiple frequencies

Example 7 in Table 2 has two frequencies present in the data. Both BSA and FFT show these two peaks in the resulting plots. Although BSA can be used in this manner with a one-dimensional *ω *to scan through frequency space and estimate the number of frequencies in the data and their location, if more than one frequency is present, the model should be extended to reflect this. Without this extension the integration procedure around a single point is not well suited, so we employed Nested Sampling to compute the marginalisation in these cases. For the extension approach, when the posterior probability over ***ω ***= {*ω*_1_} reveals two strong frequencies, then a better model would be ***ω ***= {*ω*_1_, *ω*_2_}. For example, Additional file 3, Figure S3, shows BSA and FFT results for a test case that includes higher harmonics which give rise to multiple peaks in the log *P *plot. If more than one peak in the resulting posterior probability emerges, then the model can be extended further. One peak in the posterior probability over the number of modelled frequencies signifies that the correct number of frequencies has been captured.

As another example, Additional file 4, Figure S4, shows the result of a two-frequency search. The BSA posterior probability distribution is now two-dimensional with a peak at the two correct frequencies (Additional file 4C, Figure S4C). The FFT results are also shown (Additional file 4B, Figure S4B).

Additional file 5A, Figure S5A, shows a time series with a high noise level and two very close frequencies of 0.498 and 0.505 rad/s. FFT cannot distinguish them and shows only one peak (Additional file 5B, Figure S5B). BSA breaks the resolution and precision limitations inherent to FFT by introducing a continuous probability distribution instead of the fixed number of points and can therefore sample the posterior more finely in areas of high probability. This approach gives rise to a high-resolution probability plot in which two distinct frequencies emerge (Additional file 5D, Figure S5D). The peaks have a larger variance at this local level, but the qualitative information of two underlying frequencies is revealed.

**Figure 2:**
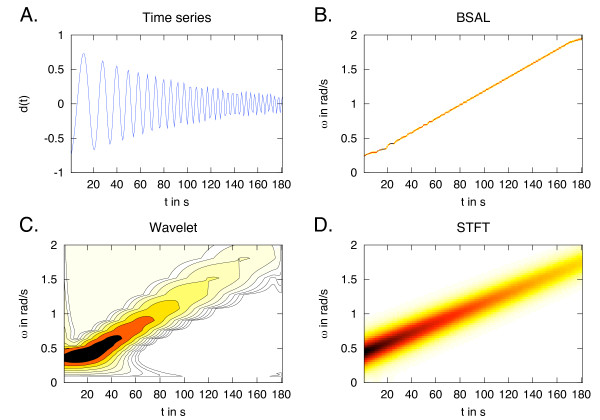
**Frequency changing over time**. A: A time series with a changing frequency, simulated from  with *ω*_1 _= 0.25 rad/s. B: The posterior probability distribution for the estimated frequency using a local BSA, BSAL, with a window size of 20. The distribution is so narrow that it resembles a sharp line in the plot. C: The wavelet power spectrum using a Morlet mother wavelet [37], with a lower cut-off at *ω *= 0.1 rad/s in the spectrum. The wavelet reproduces the correct result but with a broad distribution of frequencies. D: The STFT result with a window size of 50, and an overlap of 40, is similar to the wavelet results.

**Figure 3:**
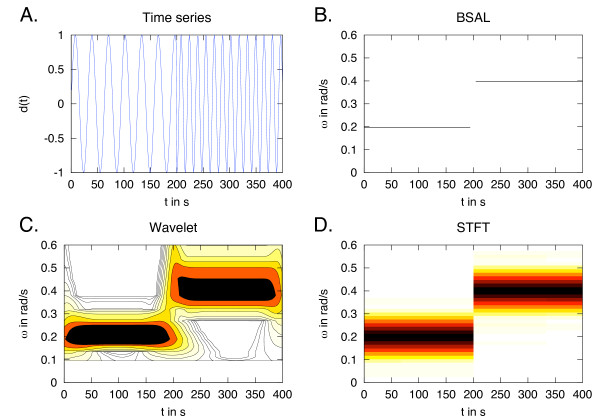
**One sharp frequency change**. A: A time series with a sharp change in frequency half way through the observed time frame, simulated from *d*(*t*) = sin(*ωt*) with *ω*_1 _= 0.2 rad/s and *ω*_2 _= 0.4 rad/s. B: The posterior probability distribution for the estimated frequency using a local BSA, BSAL, with a window size of 10, which is one third of the number of data points in a period of the first frequency. Nevertheless, the resulting distribution is so narrow that it resembles a sharp line. C: The wavelet power spectrum using a Morlet mother wavelet [37], with a lower cut-off at *ω *= 0.1 rad/s in the spectrum. D: The STFT power spectrum, window size of 100 and overlap of 5. Both the wavelet and STFT results gives the correct answer but with high variance.

To develop BSA further, we used windowing of the time series to compute the posterior probability distribution of ***ω ***at each time point. We call this BSA Local (BSAL). The robustness and negligible peak broadening of BSA with fewer time points allows for this windowing to proceed without the introduction of artefacts due to truncation. This local BSA captures changes in frequency, as shown in Figure 2B and Figure 3B. The BSAL was compared to Short-Time Fourier Transform (STFT)(Figure 2D and Figure 3D), which is a windowed Fourier Transform, and to wavelets (Figure 2C and Figure 3C). For the wavelet power spectrum a Morlet mother wavelet was used [37]. The advantages of BSAL are that it remains within the same BSA framework, has high accuracy, and does not require pre-processing of the data.

#### Non-harmonic oscillations

**Figure 4:**
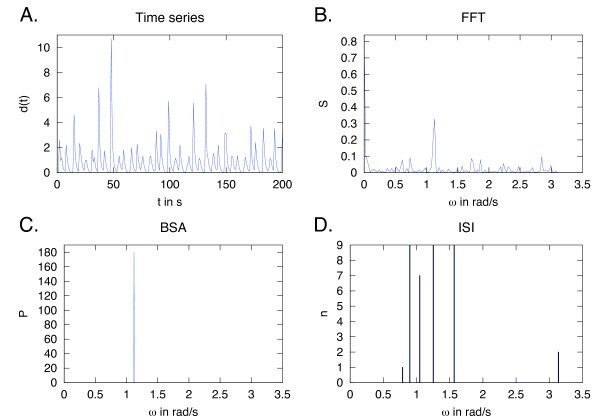
**Near-periodic oscillations**. A: A near-periodic time series simulated from a set of ODEs describing Ca^2+ ^oscillations in animal cells [38]. In the y-axes, *n *stands for the number of interspike intervals (ISI) in the ISI plot, *P *for probability in the BSA plot and *S *for spectral power in the FFT plot. The ISI plot show several intervals present (D), but both FFT (B) and BSA (C) identify a frequency around *ω *= 1.2 rad/s.

BSA results for oscillations with a non-harmonic shape are superior to the FFT. It highlights an essential difference in the two methods since biological data is often repetitive, but with a wide range of oscillatory patterns. To demonstrate this further, Figure 4A shows a time series simulated from an ordinary differential equation (ODE) model of cellular calcium (Ca^2+^) signals [38]. Such time series presents two potential problems: the time series is chaotic and thus not perfectly periodic, and the signal shape is non-harmonic. The calculation of interspike intervals (ISI) of the time series show that multiple intervals are present (Figure 4D). The highest peak of the FFT plot (Figure 4B) suggests that the entire time series is one period, while BSA suggests a strong angular frequency around 1.2 rad/s (Figure 4C). The BSA suggestion is similar to the second FFT peak.

This highlights the differences between frequencies in the data and spike intervals. ISI are a common way of characterizing spike data, however, multiple ISI need not correspond to multiple frequencies in the data. Of the four strong ISI shown here, both BSA and FFT identify only one of these as a regular period.

#### Summary

After extensive test cases we find that BSA delivers superior results in cases where the FFT assumptions are too constraining, most notably in the five cases above. BSA is a flexible method allowing the underlying hypothesis to be changed depending on the focus of the analysis, and to directly compare the validity of different hypotheses. It can handle non-uniformly sampled data and has no need for pre-processing procedures. The price of these superior results comes at a computational cost that ranged from tens to hundreds of seconds for the examples shown here.

### Calcium spiking data

The first biological data set comes from intracellular signalling in plant-microbe interactions. Symbiotic bacteria induce calcium oscillations, called Ca^2+ ^spiking, in legume root cells (for a review, see [39]). These are non-stationary and often noisy time series, causing problems in identifying periodicity. One hypothesis for signal transduction in this system is via frequency encoding [40], so concluding whether there is underlying periodicity, and at what frequency, is of great interest.

**Figure 5:**
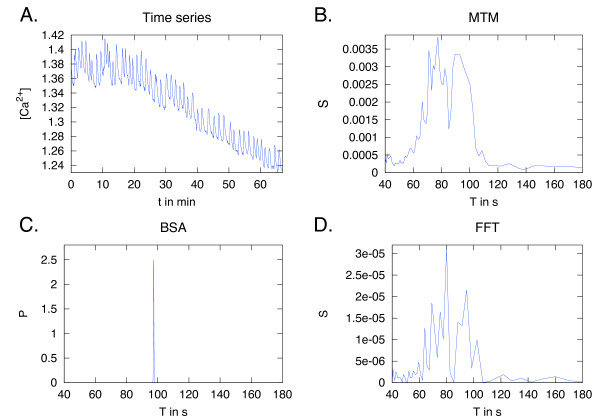
**Example results of data from calcium spiking**. A: Time series of Ca^2+ ^oscillations measured in a *M. truncatula *root hair cell in response to a bacterial signal molecule. The data is a relative ratio of the fluorescence of the Ca^2+ ^indicator dyes Oregon Green and Texas Red, showing changes in concentration, [Ca^2+^]. The measurements are taken with 5 s intervals. BSA identifies one strong frequency at a period, *T*, of about 100 s (C), while MTM (B) and FFT (D) deliver a broad spectrum.

The Ca^2+ ^spiking has background trends present due to fluorescence bleaching and cell movements, which are assumed to be unrelated to the underlying signal in the cell. Therefore, accounting for the background functions plays a key role in the analysis. Example time series are shown in Figure 5A. Nine spiking cells from the model legume *Medicago truncatula *were analysed for an underlying period. The data is obtained by microinjecting a root hair cell with the calcium indicator dyes Oregon Green (responds to Ca^2+^) and Texas Red (non-responsive), and exposing the plant to the bacterial signal molecule that induces the oscillations. The data is a ratio of the fluorescence from the two dyes, showing changes in Ca^2+ ^concentrations. The data has been published in [41].

The FFT of the Ca^2+ ^data results in a very broad periodogram, due to multiple frequencies and high noise levels (Figure 5D). Also, the spiking produces a non-harmonic signal, which might be another problem for the FFT. For comparison, we also present results from the multitaper method (MTM). The MTM is a non-parametric method of spectral analysis that uses tapers to minimize the variance in the power estimate, and is targeted at short and noisy time series [42]. The MTM results were very similar to the FFT (Figure 5B). These periodograms do not address the question of interest: Is there a key period in the Ca^2+ ^signal? In the BSA analysis (Figure 5C), the Ca^2+ ^spiking data used the Legendre background functions to an expansion order of 1-2, depending on the individual trace. Nested Sampling was used to compute the evidences. Frequencies with high probabilities were picked out, but varied in the interval of approximately 50-120 s (Table 4). However, the strongest periods were in the interval of 75-100 s. If periodicity plays a role in the signal transduction of this system, then the key period should be in this interval. The signal-to-noise ratios were relatively high, between 100-200, possibly as a consequence of including several background functions.

**Table 4:** BSA on calcium data

Cell	BSA Period ± *σ *(s)	BSA-NS Period ± *σ *(s)
1	97.4 **± **0.23	97.3 **± **0.15
2	80.9 **± **0.63	75.2 **± **10.1
3	74.6 **± **0.19	74.6 **± **0.85
4	123.8 **± **0.16	124.2 **± **1.18
5	88.9 **± **0.22	123.9 **± **0.61
6	74.6 **± **0.21	113.7 **± **16.16
7	121.9 **± **0.22	146.1 **± **21.53
8	74.4 **± **0.92	75.2 **± **2.69
9	48.2 **± **0.3	64.5 **± **13.94

Analysed calcium oscillations in *M. truncatula *root hair cells in response to bacterial symbionts. The BSA shows strong underlying signals in the data, but the cell-to-cell variability is high. BSA and the BSA-NS disagree when there are multiple peaks present, as can be seen by the large standard deviation, *σ*, of the BSA-NS average period estimate.

### Circadian data

The second biological data set shows gene expression of so-called clock genes. Many processes in plants follow a circadian rhythm (for reviews see e.g. [43] or [44]). A number of genes in *Arabidopsis thaliana *have been shown to regulate circadian rhythms, and time series of RNA levels show how these clock genes are expressed in cycles [45]. Time series with only a couple of cycles are common in biology and provide another suitable test case.

**Figure 6:**
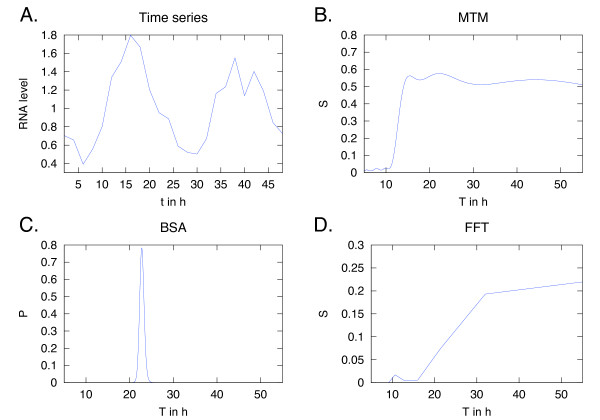
**Example results of data from a clock gene's RNA levels**. A: RNA levels of a clock gene, *TOC1*, in a *fri;flc *background of *A*. *thaliana *seedlings. The data points have 2 h intervals and consist of two averaged biological replicates. The values are normalised to the average of the housekeeping gene *ACTIN2 *in wild type. BSA gives a clear result with a period, *T*, close to 23 h (C), while MTM (B) and FFT (D) struggle with the short time series and give no clear result.

For these circadian rhythms, we chose to analyse RT-PCR data from four clock genes in two genotypes of *A. thaliana*. The plants are either wild type, *FRI;FLC*, or mutants, *fri;flc*, of the genes *FRI *and *FLC*. The RNA was extracted from seedlings, and each time series is an average of two biological replicates. An example of the RNA levels of a clock gene is shown in Figure 6A. The data has been published in [45]. FFT on the RNA levels of these clock genes did not give any clear periods, either having only a vague peak or none at all (Figure 6D). This is caused by the FFT's dependence on the length of the time series, which in this case was only 1-2 cycles. The MTM method had more of a peak in the 20-25 h period, but still lacking in precision (Figure 6B). BSA on the other hand provides a clear peak close to 23 h (Figure 6C), consistently for all eight time series (Table 5). Nested Sampling was used to compute the evidences. The assigned probabilities are relatively low, but the signal-to-noise ratios were between 2-4, and similar probabilities were obtained using simulated data with few data points and high noise levels. The period peaks are very stable over all the time series, and suggests a probable period which is unaffected by the mutations (*fri;flc*). This is in agreement with the original conclusions of the experiment [45].

**Table 5:** BSA on circadian data

Gene	Genotype	BSA Period ± *σ *(h)	BSA-NS Period ± *σ *(h)
*TOC1*	*fri;flc*	22.75 **± **0.18	22.58 **± **0.43
*TOC1*	*FRI;FLC*	23.36 **± **0.20	23.26 **± **0.42
*CCA1*	*fri;flc*	23.58 **± **0.15	23.67 **± **0.94
*CCA1*	*FRI;FLC*	23.98 **± **0.16	24.23 **± **0.72
*GI*	*fri;flc*	22.39 **± **0.14	22.54 **± **0.86
*GI*	*FRI;FLC*	23.41 **± **0.16	23.61 **± **0.83
*LHY*	*fri;flc*	23.84 **± **0.16	23.82 **± **1.54
*LHY*	*FRI;FLC*	25.74 **± **0.19	24.03 **± **1.23

The BSA results of RNA levels of four so-called clock-genes in *A. thaliana*, measured in two different genotypes. The plants are either wild type, *FRI;FLC*, or mutants, *fri;flc*, of the genes *FRI *and *FLC*. All eight genotypes displayed oscillations in RNA levels with a period of approximately 23 hours. Both BSA methods identify similar periods.

## Conclusions

Bayesian inference offers a powerful way of analysing biological time series. Despite the undisputed value of Fourier theory, there are cases when the necessary requirements for its optimality for time series analysis are not met. This is a consequence of the underlying assumptions of a Fourier Transform, causing it to work optimally only for uniformly sampled, long, stationary, harmonic signals that have either no or white noise. In biology these requirements are rarely fulfilled, requiring pre-processing of the data, such as noise reduction and detrending techniques, with the risk of convoluting the signal and losing valuable information.

By placing the problem of frequency extraction in the framework of Bayesian inference, the known and well-documented problems of Fourier analysis can be overcome. This approach also breaks the resolution and precision limitations inherent to the FFT by introducing a continuous probability distribution instead of the fixed number of points maintained by the discrete Fourier Transform. As we demonstrated here, BSA coupled with automated model development can give superior results to the FFT when faced with short, noisy time series, non-stationarity and non-harmonic signals. The suggested automated model development worked well in our hands but must be used with caution in practice as the approach is not guaranteed to find a global optimum in model space. Alternate models should be explored and compared using posterior probability ratios or approximations thereof. We found Nested Sampling [14] to provide a powerful means of estimating evidences for cases in which a single peak could not be identified. Other MCMC techniques such as simulated annealing running in parameter exploration mode or standard Metropolis-Hastings algorithms offer attractive alternatives [33].

BSA calculates signal-to-noise ratios, provides parameter precision estimates, and can handle high noise levels as well as background trends and therefore has no need for pre-processing. More importantly, the Bayesian framework offers flexibility in the underlying model and enables direct comparison of hypotheses. The work presented here is a merely a first step in this direction. We have employed conservative priors (uniform, Jeffreys, Gaussian) that make an analytical treatment tractable but in some cases more information could warrant a different choice of prior that might require substantial alternations to our approach to handle the numerics of marginalisation.

There are many known examples in biology in which oscillations play a key role and methods for their detection will be of value, especially in cases where subtle differences are of importance and for short, noisy time series. In the presented examples, we demonstrated the improvements that can be gained from employing this approach. Although in these cases, the biological conclusions would not have changed, one can envision scenarios in which a higher accuracy in frequency detection may allow subtle changes to be detected, which may otherwise have been swamped by noise and less powerful techniques. We believe that the presented methodology offers an attractive alternative to other approaches and will be a useful addition to the toolbox of systems biologists.

## Methods

All programming was done using Octave [46], which is freely available and compatible with the widely used MATLAB^®^. The Octave code is freely available from the authors upon request.

### FT

The DFT was computed using the *fft *function in Octave. The results are presented in a power spectrum, to analyse which component carries the most power. This is also know as a periodogram , i.e. the squared absolute value of the FFT of the data *d*, normalised over the number of data points *N *[2]. For time series with strong trends, detrending was done before the FFT, using the moving average method [47].

There are a number of sophisticated FT methods beyond the standard FFT, developed to avoid specific problems. For example, we also present results from the multitaper method (MTM), short-time Fourier Transforms (STFT) and wavelet analysis. For the MTM, only the MTM spectrum is presented, but it should be noted that the Singular Spectrum Analysis - MultiTaper Method (SSA-MTM) toolkit provides additional features such as significance levels of the frequencies, relative to the estimated noise levels [42]. The STFT power spectrums were computed with the *specgram *function in Octave. The wavelet results were computed using software provided by Dr. C. Torrence and Dr. G. Compo, and is available online http://atoc.colorado.edu/research/wavelets/. This wavelet software also provides additional tools such as significance levels [37].

### BSA

**Figure 7:**
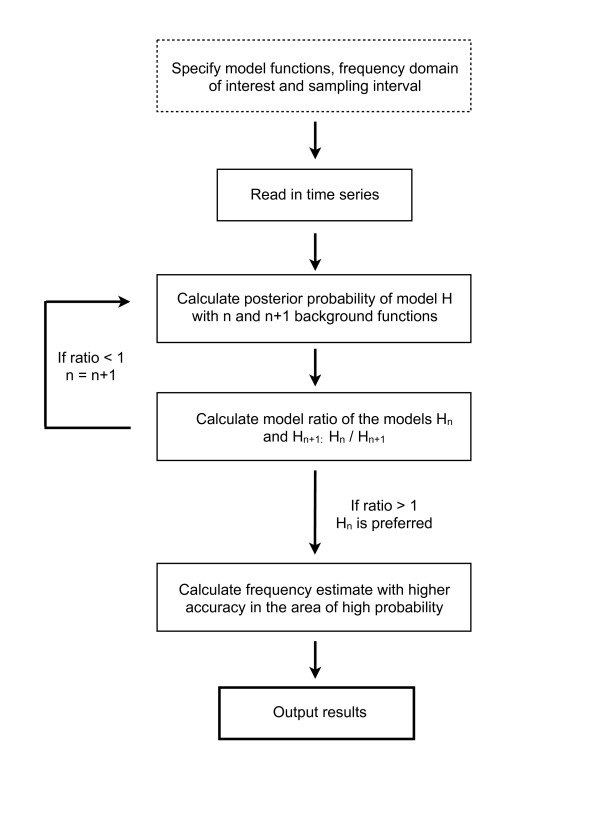
**BSA and automated background function determination**. Flowchart of the automated model development procedure for BSA. We point out that the proposed method for detecting the best number of background functions may give rise to local rather than global solutions for complex background trends and/or poor choices of background basis functions.

A flowchart of the BSA code is shown in Figure 7. The first step is to specify appropriate model and background functions. We employed sines and cosines as model functions (*ψ_j _*in equation (3)), and Legendre polynomials as background functions (*ζ_j _*in equation (4)). Legendre functions are convenient as they form a basis that can be scaled to be orthogonal over the time domain and offer a level of detail that increases with expansion order. The software, however, will attempt to orthogonalise any given set of functions over the range determined by the data by Cholesky decomposition, so other functions can be employed.

The next step is to specify the frequency domain of interest. This domain is then sampled with a chosen interval, and the posterior probability is computed at each frequency. Since the ***ω ***values are sampled over the frequency domain of interest with a chosen interval, the most probable frequency from this set may have a close neighbour with even higher probability, but which fell between sampling points. To avoid this, the Nelder-Mead optimisation technique was used to find the maximum of equation (10) [48]. Subsequently, the area surrounding this peak is finely sampled, to achieve a better representation of the posterior probability distribution of ***ω***. The number of maxima should be checked and if proceeding with a multimodal distribution, an MCMC technique such as Nested Sampling should be used instead of the described marginalisation method. The outputs from the BSA algorithm are the posterior probability distribution of ***ω***, a signal-to-noise ratio distribution and a power spectrum.

## Competing interests

The authors declare that they have no competing interests.

## Authors' contributions

RJM and GEDO conceived the experiments. RJM and EG developed and implemented the code. EG performed all the tests and analyses. EG and RJM wrote the paper. All authors read and approved the final manuscript.

## Supplementary Material

Additional File 1 **Time series with background trend**. Time series including a background trend, simulated from *d*(*t*) = sin(*ωt*) - 0.005*t*^2 ^+ *e*, with *ω *= 0.5 rad/s, sampled with 1 s intervals to give 200 points. The noise level, *e*, is 0.1 which corresponds to 10%. Click here for file

Additional File 2 **Short time series**. A: A short time series simulated from *d*(*t*) = sin(*ωt*) + *e*, with *ω *= 0.5 rad/s, and sampled with 1 s intervals to give 20 points. The noise level, *e*, is 0.1 which corresponds to 10%. B: FFT results. The y-axis is the spectral power, S. C: BSA result. P denotes the posterior probability. The BSA estimate of *ω *is correct, and has considerably less spread than the FFT estimate. Click here for file

Additional File 3 **Higher harmonics**. A: Time series with higher harmonic frequencies, simulated from *d*(*t*) = sin(*ωt*) + sin(3*ωt*) + sin(5*ωt*), with *ω *= 0.1 rad/s, sampled with 1 s intervals to give 200 points. B: FFT results. D: log(Probability) plot of the BSA results. Both BSA and FFT show three strong peaks in *ω*. Depending on the length of the series, the truncation, and sampling interval not all peaks will result in an equal probability. C: Probability plot of the BSA results. In the current case, the question of which single frequency is the most probable results in the selection of the 3*ω *frequency. Click here for file

Additional File 4 **Multiple frequencies**. A: A time series containing two distinct frequencies, simulated from *d*(*t*) = cos(*ω*_1_*t*) + cos(*ω*_2_*t*), with *ω*_1 _= 0.3 rad/s and *ω*_2 _= 0.5 rad/s, sampled with 1 s intervals to give 250 points. B: FFT results. The y-axis shows the spectral power, S. C: BSA result. Each point in this plot has two frequencies, so only off-diagonal elements correspond to two distinct frequencies and only if both are present in the data will a high joint probability emerge. Both approaches detect the correct frequencies. Click here for file

Additional File 5 **Multiple close frequencies with noise**. A: A time series containing two close frequencies, *ω*_1 _= 0.498 rad/s and *ω*_2 _= 0.505 rad/s, simulated from *d*(*t*) = cos(*ω*_1_*t*) + cos(*ω*_2_*t*) + *e*, and sampled with 1 s intervals. The noise level, *e*, is 0.1, which corresponds to 10%. B: The FFT result only shows one peak due to the sampling resolution that is determined by the time domain data. In the y-axis, S stands for spectral power. C: The BSA estimate using a two frequency model. Each point in this plot has two frequencies, so only off-diagonal elements correspond to two distinct frequencies and only if both are present will a high joint probability emerge. D: Sampling in the area around the peak of high probability show that two distinct frequencies emerge in strong off-diagonal peaks. Click here for file
