# Supplementary figures and images for: Automated Bayesian model development for frequency detection in biological time series
Source: BMC Syst Biol. 2011 Jun 24;5:97. doi: 10.1186/1752-0509-5-97 (PMC3149002; doi:10.1186/1752-0509-5-97)

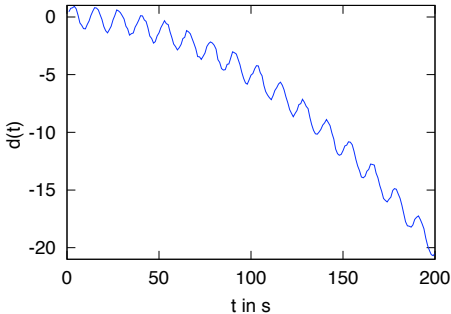

Supplement: Additional File 1 — Time series with background trend. Time series including a background trend, simulated from d(t) = sin(ωt) - 0.005t2 + e, with ω = 0.5 rad/s, sampled with 1 s intervals to give 200 points. The noise level, e, is 0.1 which corresponds to 10%. [file 1752-0509-5-97-S1.PDF]

A.

Time series

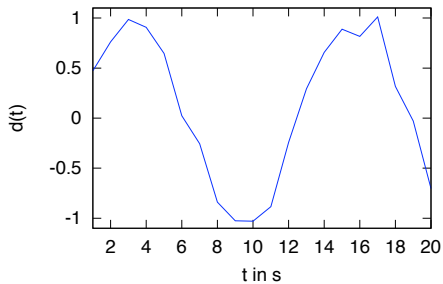

B.

FFT

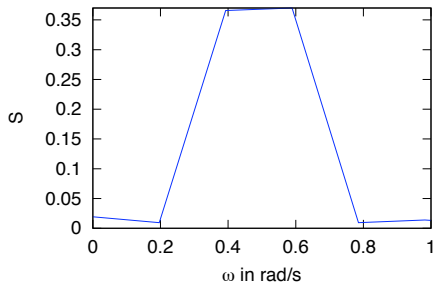

C.

BSA

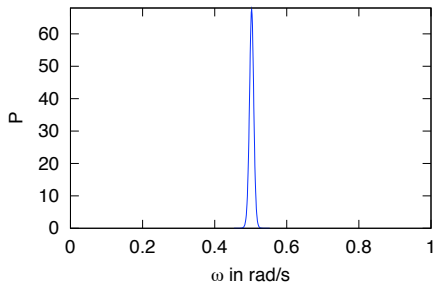

Supplement: Additional File 2 — Short time series. A: A short time series simulated from d(t) = sin(ωt) + e, with ω = 0.5 rad/s, and sampled with 1 s intervals to give 20 points. The noise level, e, is 0.1 which corresponds to 10%. B: FFT results. The y-axis is the spectral power, S. C: BSA result. P denotes the posterior probability. The BSA estimate of ω is correct, and has considerably less spread than the FFT estimate. [file 1752-0509-5-97-S2.PDF]

A.

Time series

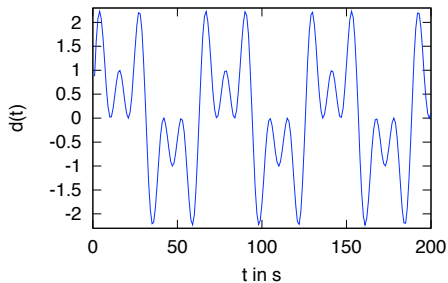

B.

FFT

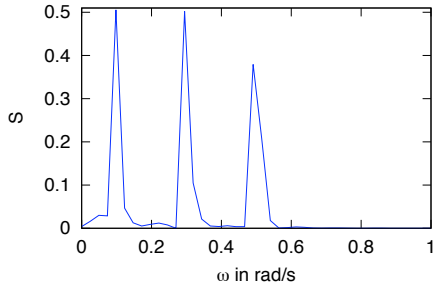

C.

BSA

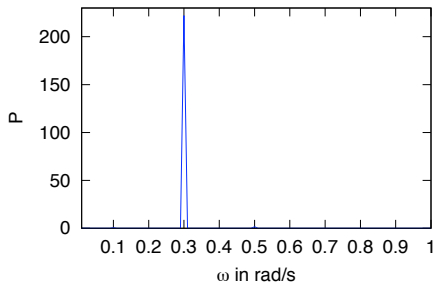

D.

BSA

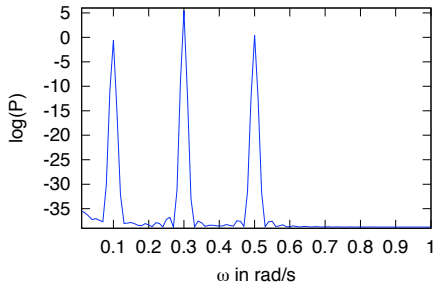

Supplement: Additional File 3 — Higher harmonics. A: Time series with higher harmonic frequencies, simulated from d(t) = sin(ωt) + sin(3ωt) + sin(5ωt), with ω = 0.1 rad/s, sampled with 1 s intervals to give 200 points. B: FFT results. D: log(Probability) plot of the BSA results. Both BSA and FFT show three strong peaks in ω. Depending on the length of the series, the truncation, and sampling interval not all peaks will result in an equal probability. C: Probability plot of the BSA results. In the current case, the question of which single frequency is the most probable results in the selection of the 3ω frequency. [file 1752-0509-5-97-S3.PDF]

A.

Time series

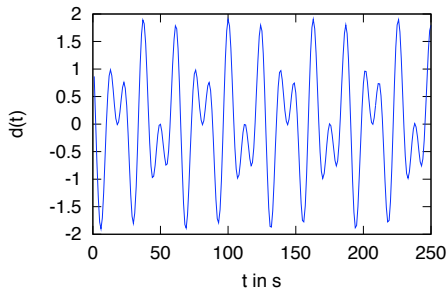

B.

FFT

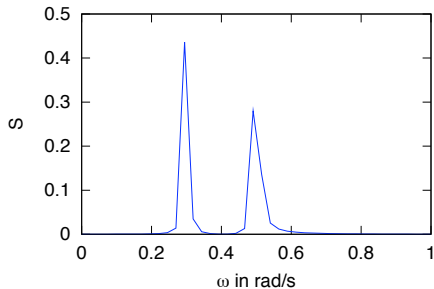

C.

BSA

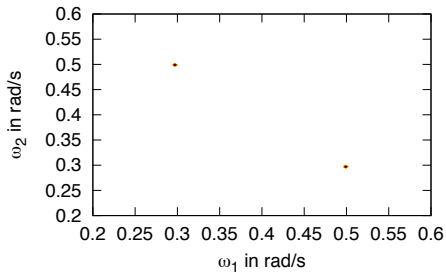

Supplement: Additional File 4 — Multiple frequencies. A: A time series containing two distinct frequencies, simulated from d(t) = cos(ω1t) + cos(ω2t), with ω1 = 0.3 rad/s and ω2 = 0.5 rad/s, sampled with 1 s intervals to give 250 points. B: FFT results. The y-axis shows the spectral power, S. C: BSA result. Each point in this plot has two frequencies, so only off-diagonal elements correspond to two distinct frequencies and only if both are present in the data will a high joint probability emerge. Both approaches detect the correct frequencies. [file 1752-0509-5-97-S4.PDF]

**A.**

Time series

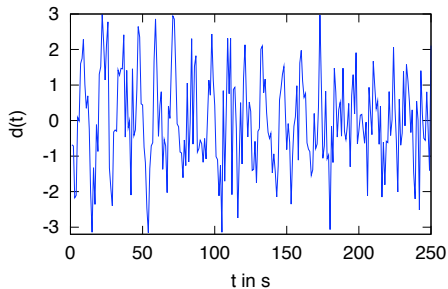**B.**

FFT

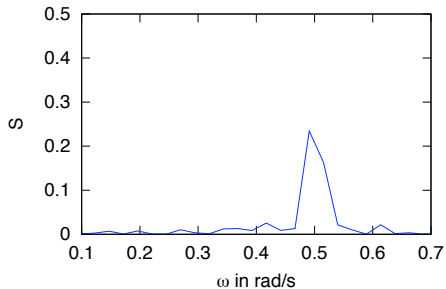**C.**

BSA

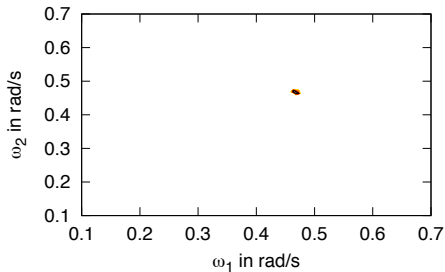**D.**

BSA

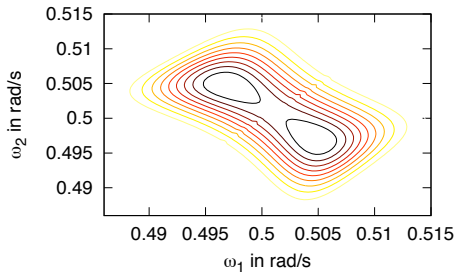

Supplement: Additional File 5 — Multiple close frequencies with noise. A: A time series containing two close frequencies, ω1 = 0.498 rad/s and ω2 = 0.505 rad/s, simulated from d(t) = cos(ω1t) + cos(ω2t) + e, and sampled with 1 s intervals. The noise level, e, is 0.1, which corresponds to 10%. B: The FFT result only shows one peak due to the sampling resolution that is determined by the time domain data. In the y-axis, S stands for spectral power. C: The BSA estimate using a two frequency model. Each point in this plot has two frequencies, so only off-diagonal elements correspond to two distinct frequencies and only if both are present will a high joint probability emerge. D: Sampling in the area around the peak of high probability show that two distinct frequencies emerge in strong off-diagonal peaks. [file 1752-0509-5-97-S5.PDF]
